# Health of Norfolk

**Published:** 1856-02

**Authors:** 


*Health of Norfolk.*—The Norfolk Herald states that that city has again assumed its wonted animation and activity in all its departments of commerce and mechanical industry, giving hopeful assurance of a recuperative energy in its population which will speedily retrieve all the pecuniary los es they may have suffered by the epidemic. Nearly all the absentees have returned, and are going about their business as usual. The few cases of sickness which lingered about the city till recently, were of persons who returned more than a month ago.

